# Association between problematic smartphone use, sleep quality, and chronotype among Sudanese medical students during armed conflict: a cross-sectional study

**DOI:** 10.3389/fpubh.2026.1816959

**Published:** 2026-07-22

**Authors:** Maria Badraldin Ali Saga, Mohammed Hammad Jaber Amin, Mohamedelmustafa Yahya Mohamed Eldouma, Mariam Salem Othman, Al-Romaysa M. Osman Khalafalla El-Haj, Hafiz Elhadi Bashar Hadi, Marafi Jammaa Ahmed, Aya Ahmed Shima, Ayat Yousif Mohammed Mohammed Saeed, Mohemed Elmahdi

**Affiliations:** 1 Faculty of Medicine, Al-Neelain University, Khartoum, Sudan; 2 Faculty of Medicine, Alzaiem Alazhari University, Khartoum, Sudan; 3 Faculty of Medicine, University of Khartoum, Khartoum, Sudan; 4 Faculty of Medicine, Alexandria University, Alexandria, Egypt; 5 Faculty of Medicine, NAHDA College, Khartoum, Sudan; 6 Faculty of Medicine, Bahri University, Khartoum, Sudan; 7 College of Medicine, University of Baghdad, Baghdad, Iraq; 8 Faculty of Medicine, Omdurman Ahlia University, Khartoum, Sudan; 9 Faculty of Medicine, Omdurman Islamic University, Khartoum, Sudan

**Keywords:** chronotype, life satisfaction, medical students, problematic smartphone use, sleep quality, smartphone addiction, Sudan

## Abstract

**Introduction:**

Mobile phones play a pivotal role in the lives of young adults and medical students, but their problematic use may negatively affect daily functioning. The ongoing armed conflict in Sudan has substantially disrupted daily routines, academic activities, psychological wellbeing, and sleep behaviors among university students. This study aimed to assess the association between problematic smartphone use and sleep quality, sleep patterns, chronotype, and life satisfaction among undergraduate medical students during the conflict period in Sudan.

**Methods:**

An analytical cross-sectional study was conducted among 387 undergraduate medical students in Sudan between September 2025 and November 2025, during an ongoing armed conflict. Data were collected using self-administered online questionnaire incorporating validated instruments: the Problematic Use of Mobile Phone (PUMP) Scale, Smartphone Addiction Scale–Short Version (SAS-SV), Pittsburgh Sleep Quality Index (PSQI), Morningness–Eveningness Questionnaire (MEQ), and Satisfaction With Life Scale (SWLS), alongside demographic variables. Descriptive statistics summarized participant characteristics and scale scores. Associations were examined using Spearman correlation analyses. Multivariable logistic regression was performed with poor sleep quality (PSQI >5) as the dependent variable. Independent variables entered simultaneously into the model included PUMP score, SAS-SV score, MEQ score, SWLS score, age, gender, and employment status. Analyses were conducted using R software, with p < 0.05 considered statistically significant.

**Results:**

A total of 387 students participated (mean age 22.1 ± 2.3 years; 69% female). Poor sleep quality (PSQI >5) was observed in 71% of participants. Smartphone addiction showed a modest but statistically significant association with poorer sleep quality (ρ =0.22, *p* < 0.001). In multivariable analysis, higher smartphone addiction scores were associated with increased odds of poor sleep quality (OR = 1.04; 95% CI: 1.00–1.07) and greater morningness preference was associated with lower odds of poor sleep quality (OR = 0.94; 95% CI: 0.91–0.96; *p* < 0.001). Employment status (OR = 2.34; 95% CI: 1.27–4.56) was independently associated with poor sleep quality (all *p* < 0.05).

**Conclusion:**

Poor sleep quality was highly prevalent among Sudanese medical students and was independently associated with smartphone addiction, evening chronotype, and employment status. These findings highlight important associations between smartphone use behaviors, chronotype, and sleep quality in conflict-affected academic settings and support the need for further longitudinal research, particularly within conflict-affected academic settings.

## Highlights

- First study to examine the association between smartphone addiction and sleep quality among Sudanese medical students during armed conflict.
- More than two-thirds (71%) of participants reported poor sleep quality during the war.
- Higher smartphone addiction scores were independently associated with poorer sleep quality.
- Morningness chronotype emerged as a significant protective factor against poor sleep (OR = 0.94).

## 1 Introduction

Mobile phones have become an essential part of modern life as a result of the 21st-century digital revolution. Mobile phones have deeply transformed daily routines, communication, and interpersonal relationships by increasing technology dependence, altering social dynamics, and potentially triggering health and family issues (1). Although digital technologies offer substantial benefits, excessive reliance on smartphones has been increasingly associated with adverse health outcomes and compulsive patterns of use (2).

Evidence suggests a strong correlation between smartphone addiction and a number of psychological issues, such as poor sleep, unsatisfactory health, and mental health problems (2–4). Medical students, in particular, represent a unique population that primarily uses smartphones to access clinical guidelines, medical literature, and other educational resources (5). Yet, excessive smartphone use was shown to be very common and strongly linked to poor sleep quality (3, 6). Moreover, students are more likely to use smartphones as a coping mechanism due to the demanding coursework, erratic schedules, and high levels of academic stress associated with medical school, a behavior that can further disrupt sleep (6). Furthermore, previous research has established that both the quantity (e.g., extended daily screen time) and timing (e.g., nocturnal use, pre-bedtime use) of smartphone engagement, besides keeping devices nearby overnight, are all linked to reduced sleep quality (7). Evening exposure to smartphone screens has been associated with circadian rhythm disruption and poorer sleep, primarily through blue-wavelength light– which suppresses melatonin, resulting in circadian disruption and poorer sleep quality (2, 8).

The connection between problematic smartphone use and sleep disturbances may be explained by a number of theoretical frameworks. According to the circadian disruption hypothesis, using a smartphone at night may affect sleep-wake regulation through exposure to blue light, cognitive stimulation, and delayed sleep timing. Overuse of smartphones has been associated with longer sleep latency and lower sleep quality, according to recent research (9, 10).

Likewise, behavioral addiction models suggest that obsessive smartphone use contributes to sleep disturbance by reinforcing maladaptive nocturnal behaviors, emotional dependency, and difficulties disengaging from digital content. (11, 12). Stress-coping models also imply that people with long-term stress may use smartphones for emotional control and diversion, which could affect sleep outcomes due to prolonged overnight use and hyperarousal (13).

The Middle East and North Africa (MENA) region, including Sudan, has a large gap between rising mental health needs and the availability of psychiatric resources (14).

In conflict-affected areas, disturbed habits, instability, displacement, and emotional distress can all have an impact on smartphone use and sleep patterns. Smartphones are frequently used for safety, emergency communication, news and other purposes. Recent research in displaced and war-affected people demonstrates that excessive exposure to upsetting digital content and persistent connectivity are related to higher psychological distress, hyperarousal, and lower sleep quality (15).

In Sudan, the ongoing armed conflict has further intensified this gap by disrupting public health systems and straining already scarce resources. In these unstable environments, smartphones may function beyond their general use for social interaction to the extent of being a ‘digital proxy' for social anxiety, (16) especially when access to psychiatric resources is severely curtailed, which may result in an exacerbation of the detrimental effects on the circadian rhythms (17). To date, the prevalence of problematic mobile phone use among Sudanese medical students, and its association with sleep quality, chronotype, and overall life satisfaction, has not been comprehensively examined.

To address these critical gaps, we conducted a study among undergraduate medical students in Sudan during a period of ongoing conflict. This unique context provides an opportunity to examine problematic mobile phone use and sleep health under conditions of heightened stress and limited resources. Accordingly, this study aimed to assess the association between problematic smartphone use and sleep quality among undergraduate medical students in Sudan. It also examined its relationships with chronotype, sleep patterns, and life satisfaction.

## 2 Materials and methods

### 2.1 Study design

An analytical cross-sectional study was conducted to assess the association between problematic smartphone use and sleep quality among undergraduate medical students in Sudan. Given the study design, causal or temporal relationships could not be inferred. Data collection took place from 15 September 2025 to 12 November 2025, using an online, self-administered questionnaire. All enrolled undergraduate medical students in Sudan were considered eligible for participation.

### 2.2 Study setting and population

The study was performed among undergraduate students enrolled in medical faculties across different Sudanese universities, both governmental and private. Data collection occurred online during a period of ongoing armed conflict, which has profoundly disrupted education and daily life in Sudan. The target population was all students officially enrolled in undergraduate medical programs at Sudanese universities. Participants were considered eligible and included if they were currently enrolled as undergraduate medical students in Sudan, and electronic informed consent was obtained from all participants before survey completion. The requirement for written informed consent was waived by the Institutional Review Board because the study involved an anonymous, minimal-risk online survey. Participants were excluded if they were not medical students or refused to participate in the study.

### 2.3 Sampling and participants

The minimum required sample size was calculated using the single population proportion formula for an unknown prevalence:


$$\begin{array}{l} n=\;Z^{2}\times P\times q÷d^{2} \end{array}$$

where:

· n = sample size,
· Z = Z-score for 95% confidence level (1.96),
· *p* = expected proportion (0.5, for maximum variability),
· q = 1–*p* (0.5),
· d = margin of error (0.05).

The calculation yielded a minimum sample of 385 participants. To account for potential incomplete responses, the survey was disseminated broadly.

Given the absence of a centralized national registry and the disruption caused by the conflict, a non-probability convenience sampling method was employed. This approach was realistic but imposes limitations, including the inability to calculate a formal response rate and the potential for selection bias, as participants were self-selected and likely over-represent students with greater digital access and engagement. The survey link was distributed via university-affiliated social media groups and student networks across multiple medical schools in Sudan. Because the survey link was distributed through overlapping student networks and social media platforms, the number of students who saw the invitation could not be accurately determined; thus, a response rate could not be calculated.

### 2.4 Data collection instrument

Data were collected using a structured, self-administered online questionnaire hosted on Google Forms. The questionnaire was designed by the research team to address the study objectives and was composed of six sections, using standardized and validated scales, each had been used in its original English version, as English is the primary medium of medical instruction in Sudan. Questionnaire sections were as follows:

1. Socio-demographic Profile (11 items): Collected information on age, gender, parental education, employment, living situation, socio-economic status, marital status, college year, and current GPA.
2. Problematic Use of Mobile Phone (PUMP) Scale: A 16-item scale assessing compulsive use, withdrawal, and functional impairment related to smartphones. Responses were recorded on a 5-point Likert scale (1 = Strongly Disagree to 5 = Strongly Agree) (12).
3. Smartphone Addiction Scale – Short Version (SAS-SV): A 10-item scale measuring addiction-like symptoms. Items were rated on a 6-point Likert scale (1 = Strongly Disagree to 6 = Strongly Agree) (13).
4. Satisfaction With Life Scale (SWLS): A 5-item global assessment of subjective life satisfaction, rated on a 7-point Likert scale (1 = Strongly Disagree to 7 = Strongly Agree) (14).
5. Morningness-Eveningness Questionnaire (MEQ): A 19-item instrument determining circadian rhythm preference (chronotype), categorizing individuals from “Definitely Evening” to “Definitely Morning” type (15).
6. Pittsburgh Sleep Quality Index (PSQI): An 18-item instrument evaluating seven sleep components over the past month to generate a global score, where a score >5 indicates “poor sleep quality” (16).

The PUMP scale assessed broader aspects of problematic smartphone use, including compulsive behavior and functional impairment, while the SAS-SV measured addiction-like symptoms. Although related, both instruments capture distinct yet complementary dimensions and were used to provide a more comprehensive assessment and enable comparison with previous studies.

### 2.5 Study variables

The primary dependent variable (outcome) was sleep quality, operationally defined as a binary variable: poor sleep quality (defined as a global PSQI score > 5) versus good sleep quality (PSQI score ≤ 5). The main independent variables of interest were problematic mobile phone use and smartphone addiction, measured by the total scores on the Problematic Use of Mobile Phone Scale (PUMP) and the Smartphone Addiction Scale-Short Version (SAS-SV), respectively. Additional secondary variables of interest included:

· Chronotype: Operationally defined as the total score on the Morningness-Eveningness Questionnaire (MEQ), with higher scores indicating a stronger “morningness” preference.
· Life satisfaction: Operationally defined as the total score on the Satisfaction With Life Scale (SWLS).

A set of socio-demographic and academic covariates were collected for descriptive purposes and to control for potential confounding. These included: age (years), gender (male/female), employment status (employed/unemployed), socioeconomic status (low/moderate/high), living situation, study year, and current GPA. It is important to note that while we captured perceived change in smartphone use attributed to the conflict, more direct measures of conflict exposure—such as displacement status or direct exposure to violence—were not collected.

Finally, a descriptive variable on perceived change in smartphone use due to the ongoing conflict was captured via a single 5-point ordinal item (“Decreased a lot” to “Increased a lot”).

### 2.6 Data analysis

Statistical analyses were conducted using R, version 4.4.3. Descriptive statistics were used to summarize the data. Continuous variables, including scores from the various scales (PUMP, SAS-SV, SWLS, MEQ, and PSQI) were presented as mean ± standard deviation (SD). Categorical variables, such as gender and derived classifications like SWLS categories, MEQ chronotypes, and PSQI sleep quality (good/poor) were summarized using frequencies (n) and percentages (%). Stacked bar charts were used to visualize the distribution of responses for the individual items on the Problematic Use of Mobile Phone Scale (PUMP) and the Smartphone Addiction Scale–Short Version (SAS-SV). The normality of continuous variables was assessed using the Shapiro-Wilk test. Because most continuous variables demonstrated non-normal distributions, non-parametric correlation analyses were prioritized. Hence, Spearman's rank correlation (ρ) was calculated to assess the strength and direction of the correlation between the scale scores. A correlation matrix and scatter plots with superimposed regression lines were generated to visualize the Spearman correlation coefficients between the scales. A *p*-value of less than 0.05 was considered statistically significant. Variables included in the multivariable logistic regression model were selected *a priori* based on theoretical relevance and prior literature. All selected variables were entered simultaneously into the model rather than through stepwise selection procedures. To identify independent factors associated with poor sleep quality, a multiple logistic regression analysis was performed. The dependent variable was poor sleep quality, defined as a global PSQI score > 5, results are presented as adjusted odds ratios (OR) with their corresponding 95% confidence intervals (CI).

Furthermore, the multivariable logistic regression model's reliability was confirmed using the Hosmer-Lemeshow test to ensure an adequate goodness-of-fit for predicting poor sleep quality.

Multicollinearity was assessed using variance inflation factors (VIFs). All VIF values were below 2, indicating no evidence of problematic multicollinearity.

### 2.7 Ethical considerations

All procedures carried out in this study were in accordance with the ethical standards outlined in the Declaration of Helsinki. Ethical approval was obtained from the Institutional Review Board of the Faculty of Medicine, Alzaiem Alazhari University(Approval No: AAU-REC-FMED-2023-042). Participation was entirely voluntary and anonymous. Informed consent was obtained electronically from all participants prior to participation. The first page of the online questionnaire presented a detailed participant information sheet, outlining the study's purpose, procedures, risks, benefits, and confidentiality measures. Informed consent was obtained electronically, participants could only proceed to the survey questions after agreeing to participate. This study was reported in accordance with the Checklist for Reporting Results of Internet E-Surveys (CHERRIES) to ensure the transparency, quality, and comprehensive documentation of the web-based data collection process. The study also adheres to the STROBE (Strengthening the Reporting of Observational Studies in Epidemiology) guidelines. Participants could withdraw at any point without any penalty. All data were stored on a protected data sheet and used solely for aggregate research purposes. No ethical concerns or limitations were identified during the study.

## 3 Results

A total of 387 undergraduate medical students participated in the study. The mean age was 22.05 ± 2.30 years. Most students were female (69%), single (96%), unemployed (79%), and living with parents or family members (90%). The mean GPA was 3.22 ± 0.51. Regarding smartphone use during the conflict, about half of participants reported increased use, while 29% reported no change. Additional demographic characteristics are presented in Table 1.

**Table 1:** Demographic characteristic of the study participants.

Characteristic	N = 387^1^
**Age (Years)**	22.05 ± 2.30
Gender	
Female	268 (69%)
Male	119 (31%)
Father's education level	
Less than high school	34 (8.8%)
High school graduate	71 (18%)
Some college	58 (15%)
2 year degree	6 (1.6%)
4 year degree	50 (13%)
Professional degree	116 (30%)
Doctorate	52 (13%)
Mother's education level	
Less than high school	39 (10%)
Some college	55 (14%)
High school graduate	107 (28%)
2 year degree	13 (3.4%)
4 year degree	58 (15%)
Professional degree	86 (22%)
Doctorate	29 (7.5%)
Marital status	
Single	373 (96%)
Married	14 (3.6%)
Employment status	
Unemployed	306 (79%)
Employed	81 (21%)
Living situation	
With parent or family	349 (90%)
On your own	20 (5.2%)
Homeless	2 (0.5%)
Other	16 (4.1%)
Socio-economic status	
Low	19 (4.9%)
Moderate	352 (91%)
High	16 (4.1%)
Study year	
1st year	40 (10%)
2nd year	66 (17%)
3rd year	59 (15%)
4th year	103 (27%)
5th year	82 (21%)
6th year	37 (9.6%)
**Current GPA**	3.22 ± 0.51
Not reported	97
How has your smartphone use changed during the conflict?	
Decreased a lot	19 (4.9%)
Decreased	65 (17%)
Same	114 (29%)
Increased	113 (29%)
Increased a lot	76 (20%)

^1^Mean ± SD; n (%).

### 3.1 Problematic mobile phone use and addiction

**Figure 1:**
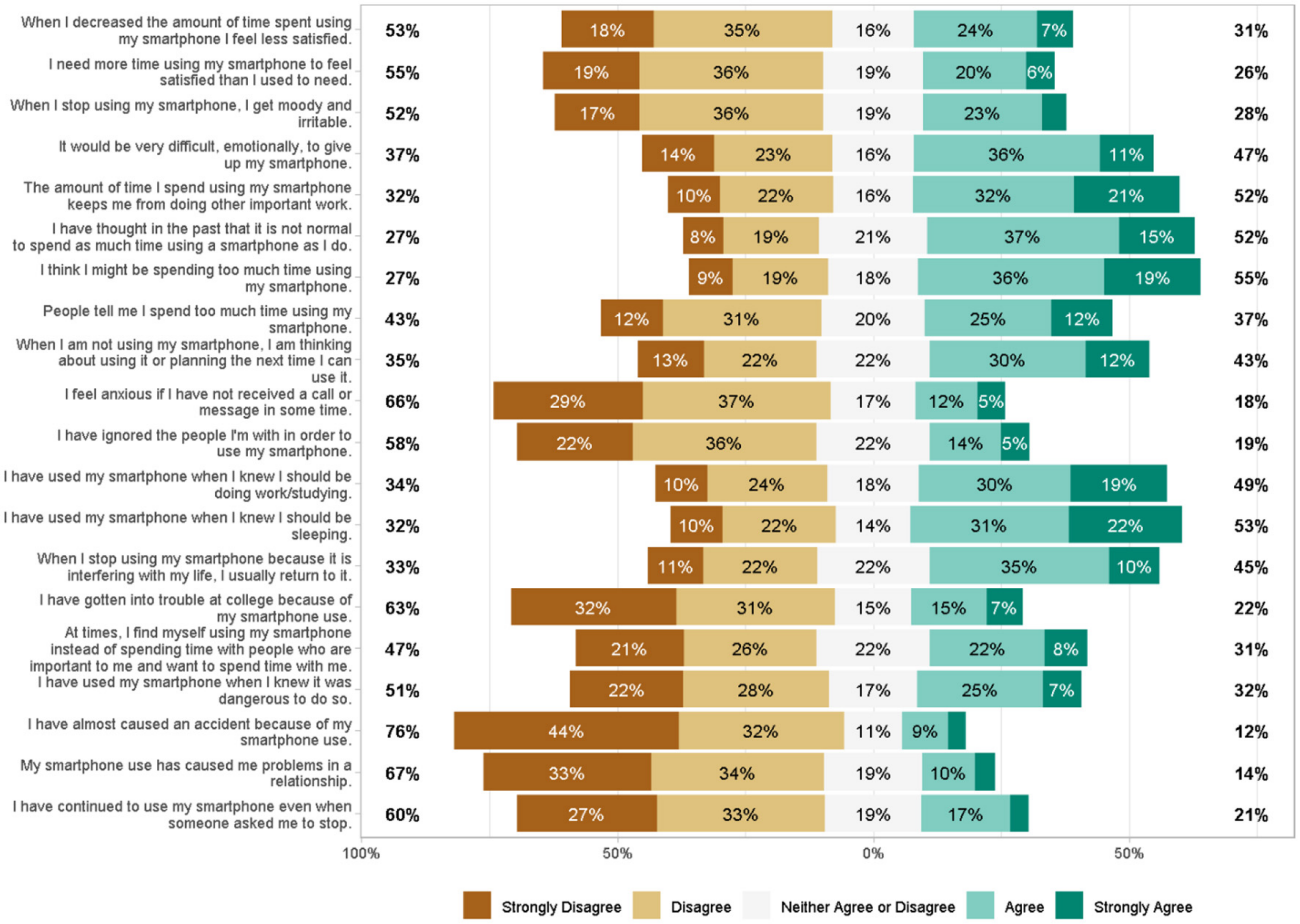
Problematic Use of Mobile Phone Scale (PUMP) questions.

**Figure 2:**
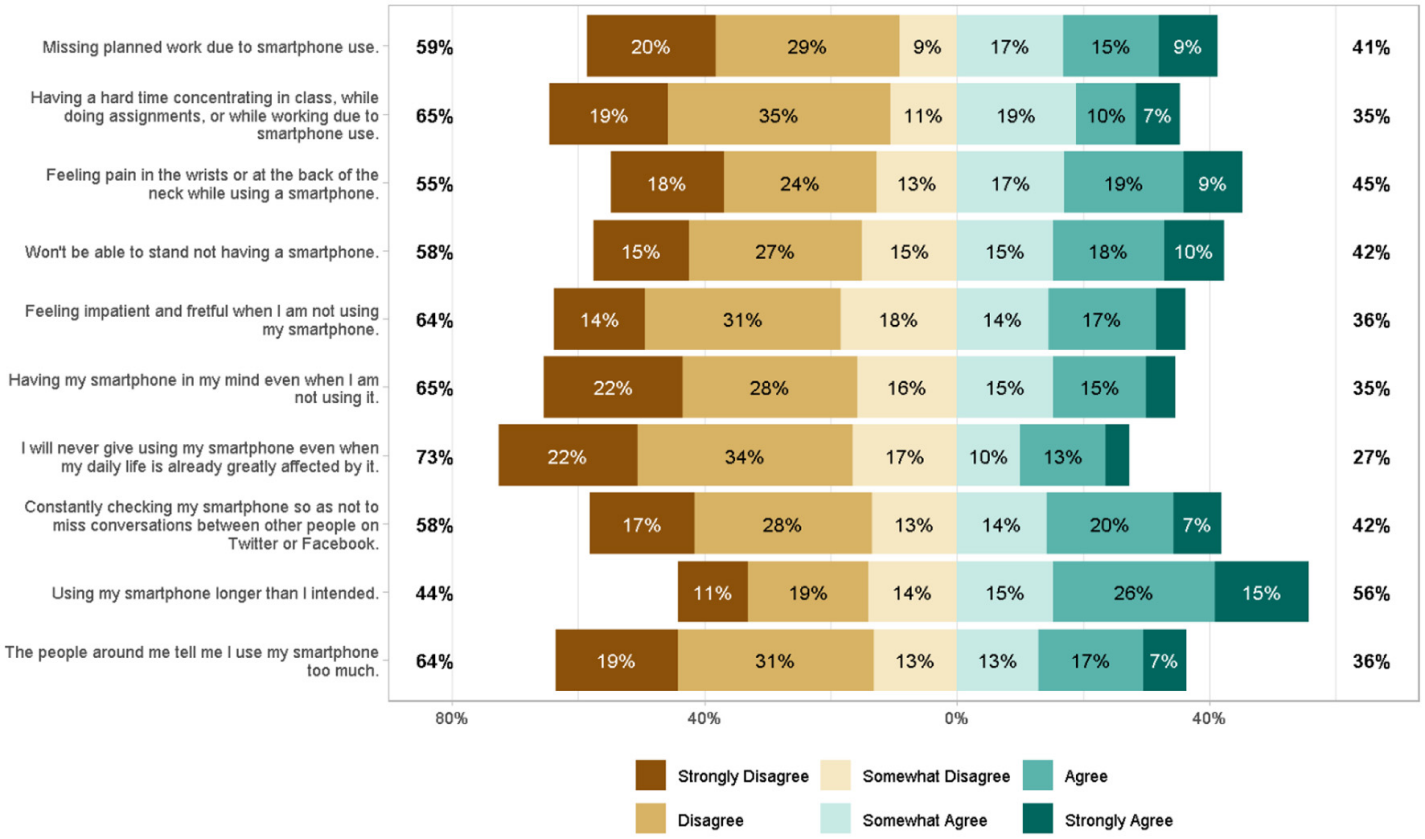
Smartphone addiction Scale – Short Version (SAS-SV) questions.

The mean score on the Problematic Use of Mobile Phone Scale (PUMP) was 56 ± 15. Figure 1 illustrates the distribution of responses to PUMP questions. A notable proportion strongly agreed with items related to excessive time spent on smartphones and difficulty controlling use (Table 2, Figure 1). On the other hand, the mean score on the Smartphone Addiction Scale–Short Version (SAS-SV) was 31 ± 11. Figure 2 displays the response distributions for SAS-SV items, highlighting concerns such as missing planned work due to smartphone use and feeling pain in the wrists or neck from usage.

**Table 2:** Descriptive statistics, normality assessment, and reliability of the scales used in the study (*n* = 387).

Characteristic	Summary^1^	Shapiro-Wilk test (*p*-value)	Cronbach's Alpha (α)
**Problematic use of mobile phone scale (PUMP)**	56 ± 15	0.566	0.91
**Smartphone addiction Scale—Short Version (SAS-SV)**	31 ± 11	< 0.001	0.89
**Satisfaction With Life Scale (SWLS)**	19 ± 7	< 0.001	0.86
SWLS categories			
Extremely dissatisfied	40 (10%)		
Dissatisfied	71 (18%)		
Slightly dissatisfied	66 (17%)		
Neutral	25 (6.5%)		
Slightly satisfied	95 (25%)		
Satisfied	73 (19%)		
Extremely satisfied	17 (4.4%)		
**Morningness-eveningness questionnaire (MEQ)**	53 ± 9	0.003	0.74
MEQ categories			
Definitely evening type	9 (2.3%)		
Moderately evening type	28 (7.2%)		
Neither type	243 (63%)		
Moderately morning type	99 (26%)		
Definitely morning type	8 (2.1%)		
**Pittsburgh sleep quality index (PSQI) global score**	6.7 ± 3.4	< 0.001	0.63
PSQI sleep quality			
Good sleep quality	111 (29%)		
Poor sleep quality	276 (71%)		
**PSQI—Subjective sleep quality**	1.15 ± 0.80		
**PSQI—Sleep latency**	1.28 ± 0.98		
**PSQI—Sleep duration**	0.75 ± 1.01		
**PSQI—Habitual sleep efficiency**	0.65 ± 1.06		
**PSQI—Sleep disturbances**	1.39 ± 0.63		
**PSQI—Use of sleeping medication**	0.37 ± 0.73		
**PSQI—Daytime dysfunction**	1.05 ± 0.83		

^1^Mean ± SD; n (%).

### 3.2 Life satisfaction, sleep quality, & sleep pattern

The mean Satisfaction With Life Scale (SWLS) score was 19 ± 7, with most participants falling within the slightly satisfied or satisfied categories. The mean Morningness-Eveningness Questionnaire (MEQ) score was 53 ± 9, and the majority of students were classified as neither chronotype. Sleep quality assessment using the Pittsburgh Sleep Quality Index (PSQI) showed a mean global score of 6.7 ± 3.4, with 71% of students classified as having poor sleep quality. The high prevalence of poor sleep quality may reflect the combined effects of academic stress, conflict-related disruption, psychological strain, and altered daily routines during the ongoing armed conflict. Among PSQI components, sleep disturbances and sleep latency demonstrated the highest mean scores. Detailed categorical distributions and PSQI component scores are presented in Table 2.

### 3.3 Reliability and normality assessment of measurement scales

Internal consistency for all scales used in the study was assessed using Cronbach's alpha. The Problematic Use of Mobile Phone Scale (α = 0.91), the Smartphone Addiction Scale-Short Version (α = 0.89), and the Satisfaction with Life Scale (α = 0.86) all demonstrated excellent to good internal reliability. However, the Morningness-Eveningness Questionnaire (α = 0.74) showed acceptable reliability. Although the PSQI demonstrated lower internal consistency in our sample (α = 0.63), this finding is consistent with previous studies in student populations and likely reflects the multidimensional structure of sleep quality, where components such as medication use and sleep duration may not strongly correlate.

Normality was assessed using the Shapiro-Wilk test. All continuous variables, except for the Problematic Use of Mobile Phone Scale, were found to be not normally distributed (*p* < 0.05) (Table 2).

### 3.4 Correlations and relationships

**Figure 3:**
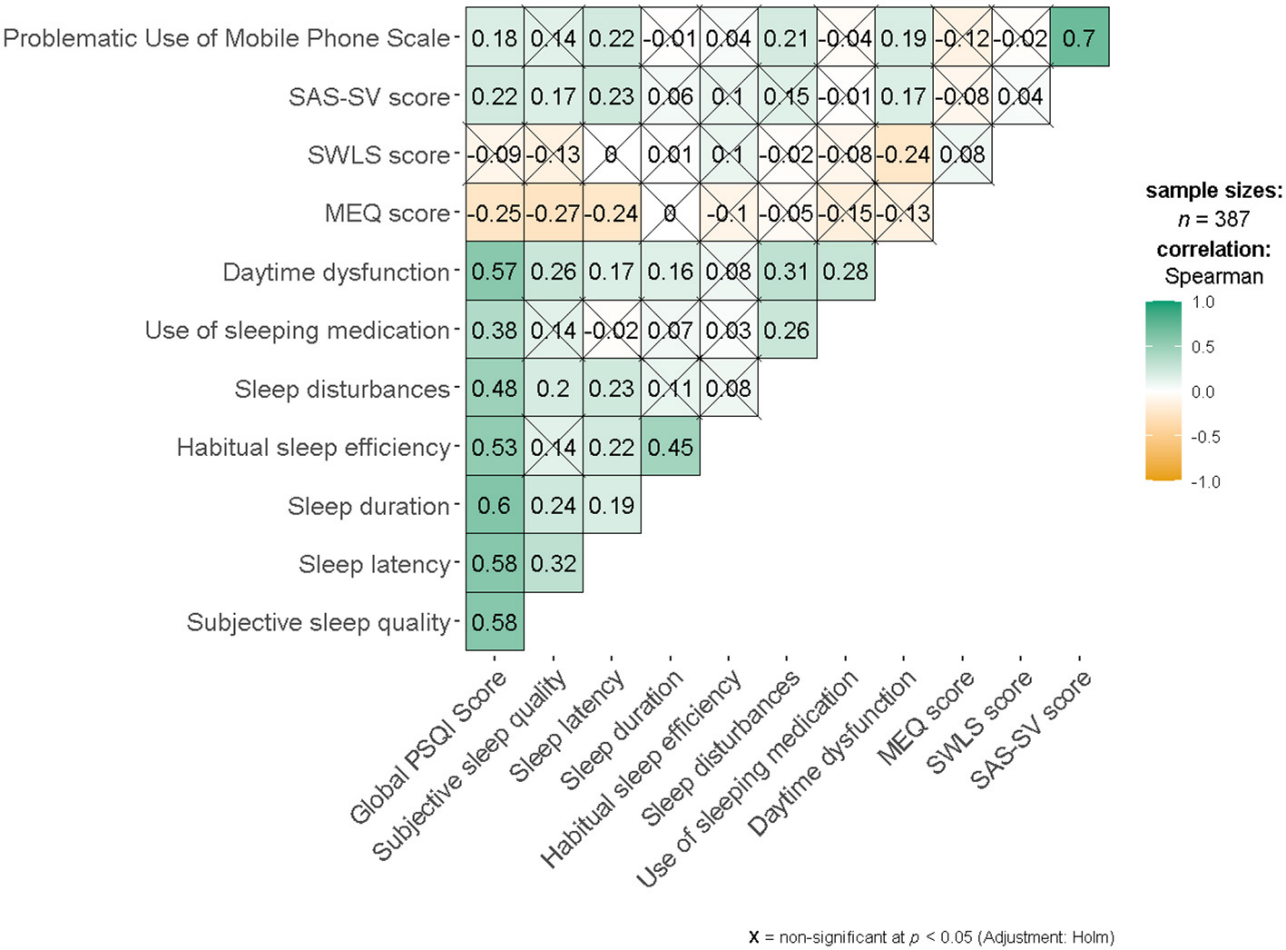
Correlation matrix showing Spearman correlation of problematic mobile phone use and addiction with Sleep quality and pattern.

**Figure 4:**
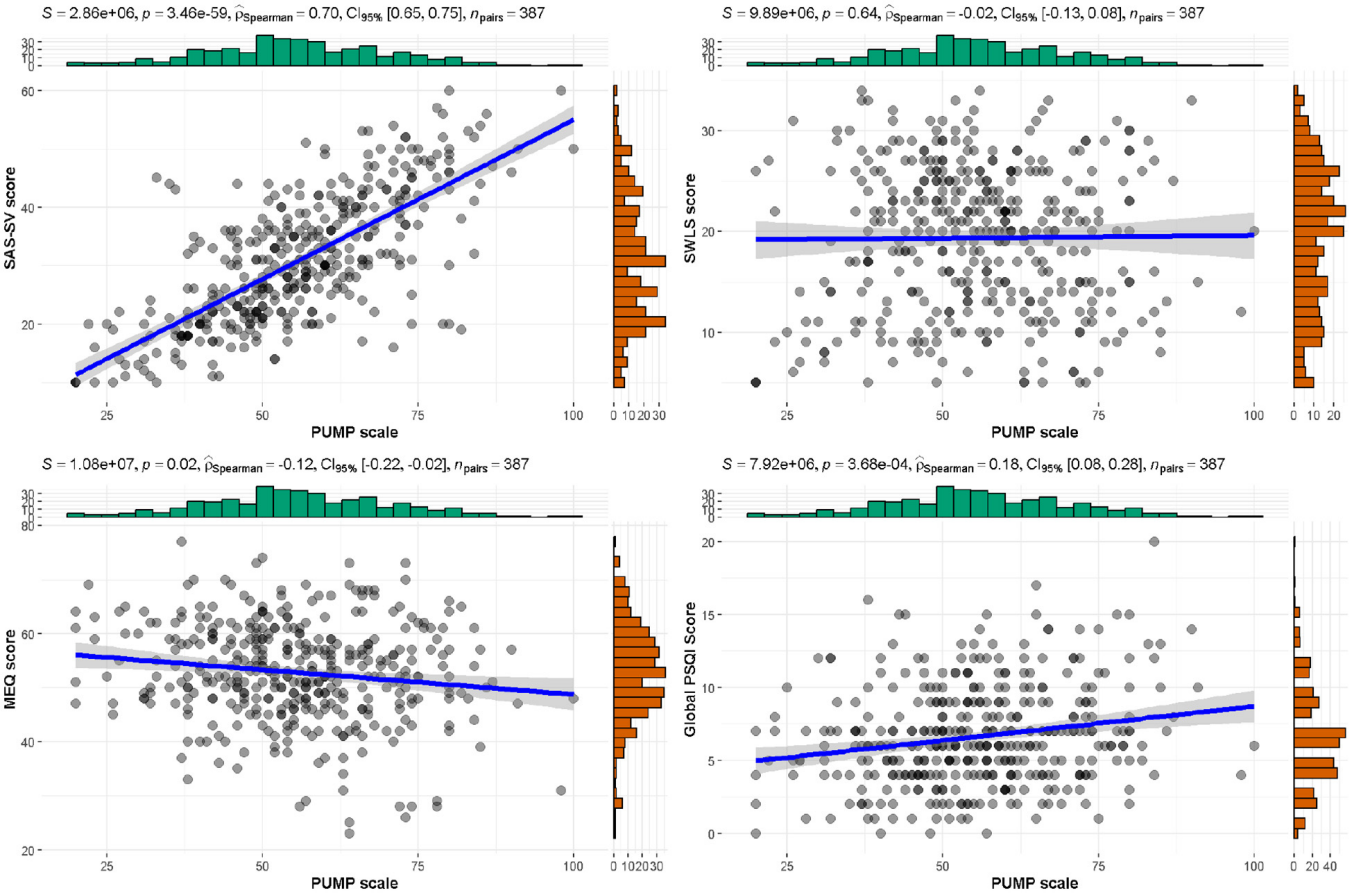
Scatter plots with Spearman correlation of Problematic Use of Mobile Phone Scale (PUMP) with Smartphone addiction Scale—Short Version (SAS-SV), Satisfaction With Life Scale (SWLS), Morningness-Eveningness Questionnaire (MEQ), & Pittsburgh Sleep Quality Index (PSQI) scores.

**Figure 5:**
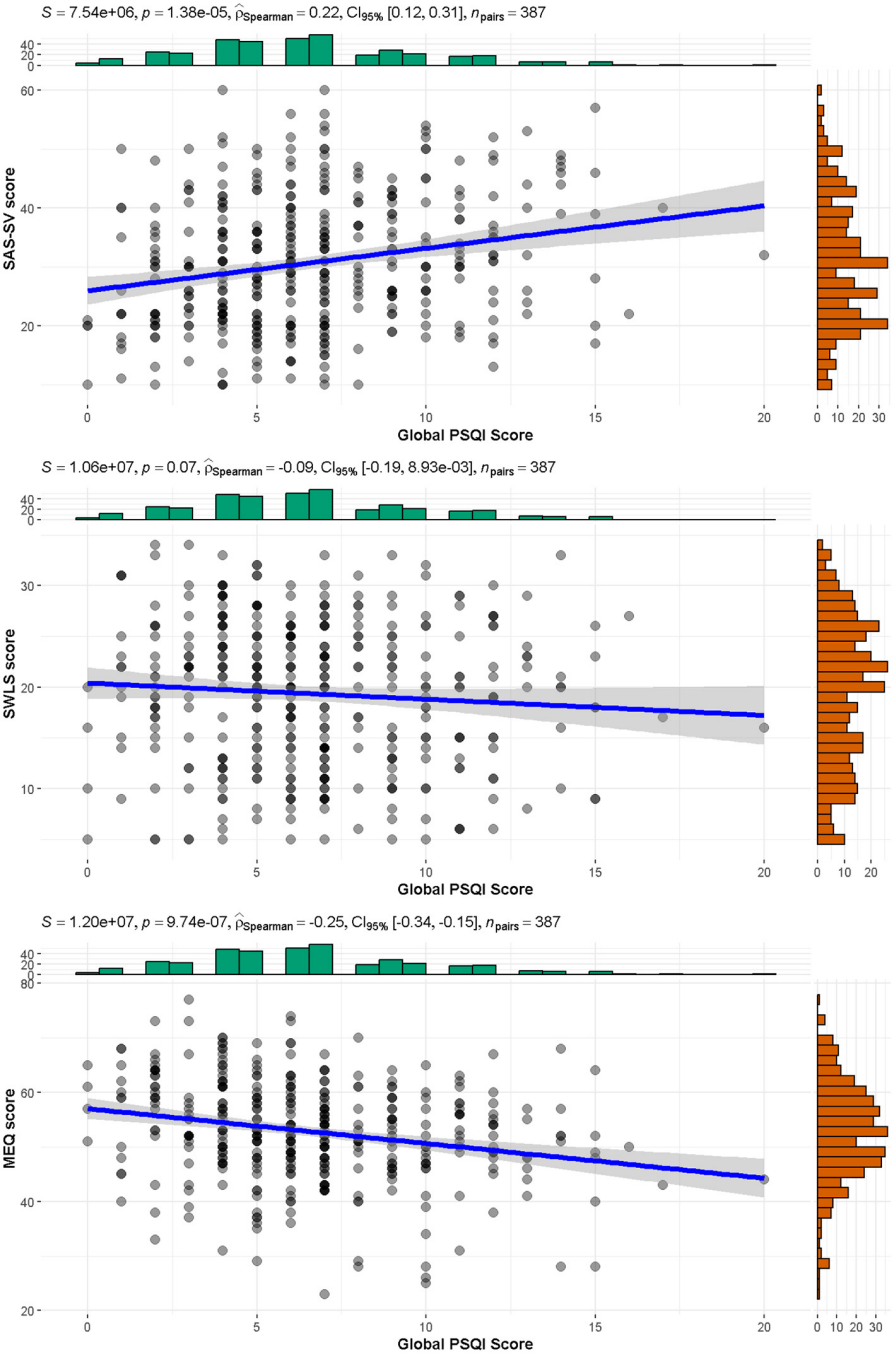
Spearman correlation of Pittsburgh Sleep Quality Index (PSQI) global score with Smartphone addiction Scale—Short Version (SAS-SV), Satisfaction With Life Scale (SWLS), & Morningness-Eveningness Questionnaire (MEQ).

**Figure 6:**
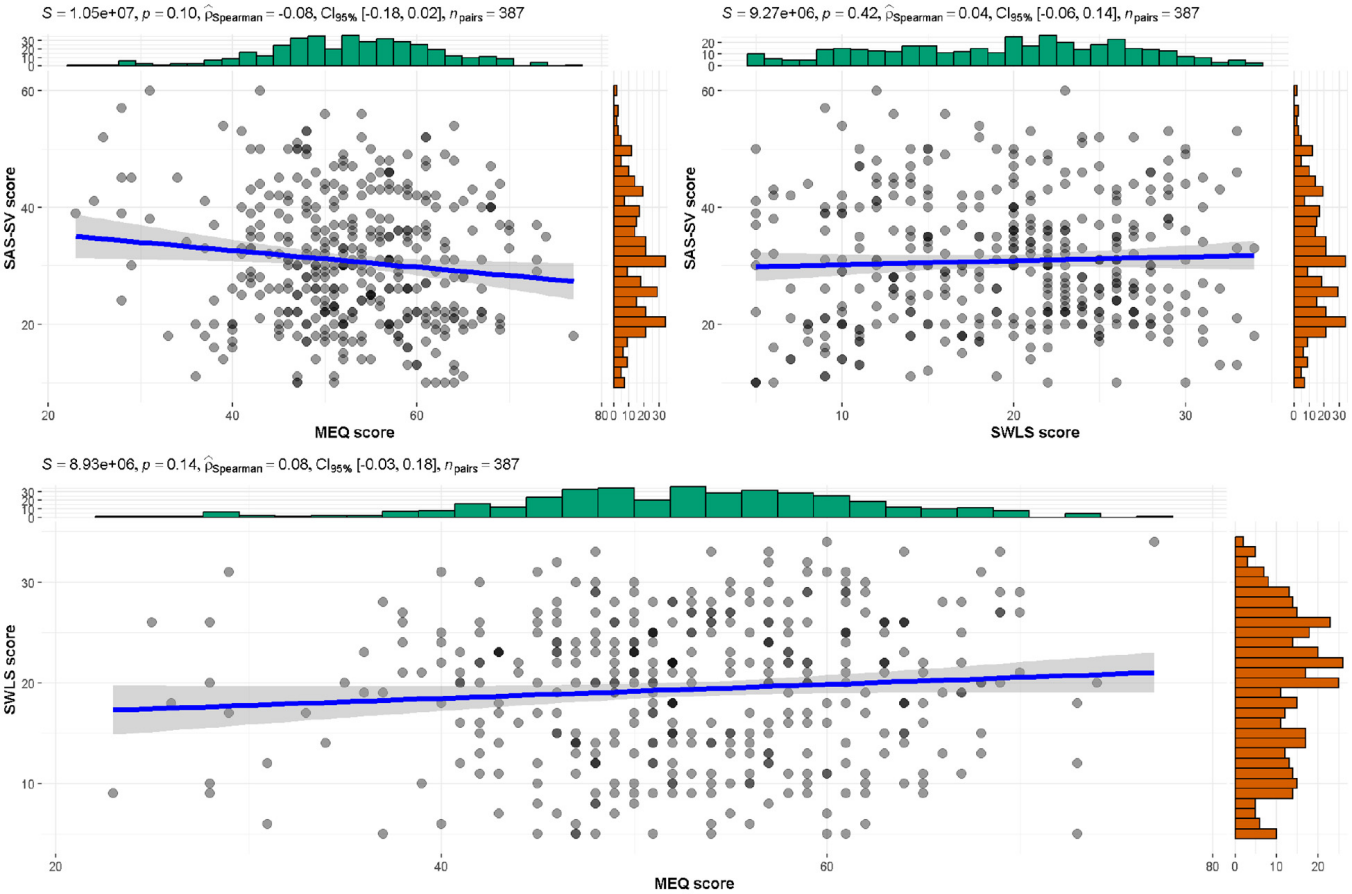
Spearman correlation of Smartphone addiction Scale—Short Version (SAS-SV), Satisfaction With Life Scale (SWLS), & Morningness-Eveningness Questionnaire (MEQ).

Spearman correlation analysis revealed low to moderate associations between problematic mobile phone use, smartphone addiction, sleep quality, and chronotype (Figure 3). Scatter plots visualization using non-parametric approach further explored these relationships (Figures 4–6). PUMP scores showed significant positive correlations with SAS-SV (ρ = 0.7, *p* < 0.001) and PSQI scores (ρ = 0.18, *p* < 0.001), alongside a negative correlation with MEQ scores (ρ = −0.12, *p* = 0.02). PSQI global scores correlated positively with SAS-SV scores (ρ = 0.22, *p* < 0.001) and negatively with MEQ scores (ρ = −0.25, *p* < 0.001). Additional analyses demonstrated a weak negative association between MEQ and SAS-SV scores (ρ = −0.08, *p* = 0.1), and a weak positive association between MEQ and SWLS scores was not statistically significant (*r* = 0.08, *p* = 0.14).

### 3.5 Predictors of poor sleep quality

A multivariable logistic regression model was used to identify independent predictors of poor sleep quality (global PSQI > 5). Both the scores of Smartphone Addiction Scale (SAS-SV) and Morningness–Eveningness Questionnaire (MEQ) as well as employment status emerged as significant predictors (Table 3). Higher scores on the Smartphone Addiction Scale (SAS-SV) were associated with modestly increased odds of poor sleep quality (OR = 1.04; 95% CI: 1.00–1.07; *p* = 0.027). In contrast, greater morningness preference was associated with lower odds of poor sleep quality: each one-unit increase in the Morningness–Eveningness Questionnaire (MEQ) score, reflecting greater morningness, was associated with a 6% reduction in the odds of poor sleep quality (OR = 0.94; 95% CI: 0.91–0.96; *p* < 0.001). Employment status was also a significant predictor, with employed participants exhibiting more than twice the odds of poor sleep quality compared with unemployed participants (OR = 2.34; 95% CI: 1.27–4.56; *p* = 0.009). Age, gender, and life satisfaction (SWLS) were not significantly associated with poor sleep quality in the adjusted model (Table 3).

**Table 3:** Multiple logistic regression model predicting poor sleep quality.

Characteristic	OR	95% CI	*p*-value
**PUMP score**	0.99	0.97, 1.02	0.6
**SAS-SV score**	1.04	1.00, 1.07	**0.027**
**SWLS score**	0.97	0.94, 1.01	0.10
**MEQ score**	0.94	0.91, 0.96	**< 0.001**
**Age (Years)**	0.93	0.84, 1.04	0.2
Gender			
Female	—	—	
Male	1.29	0.77, 2.21	0.3
Employment status			
Unemployed	—	—	
Employed	2.34	1.27, 4.56	**0.009**

CI, Confidence Interval; OR, Odds Ratio. Reference categories: Female gender and unemployed status.

OR, Odds Ratio; CI, Confidence Interval; VIF, Variance Inflation Factor. Bold values indicate statistically significant associations (*p* < 0.05).

### 3.6 Model performance

**Figure 7:**
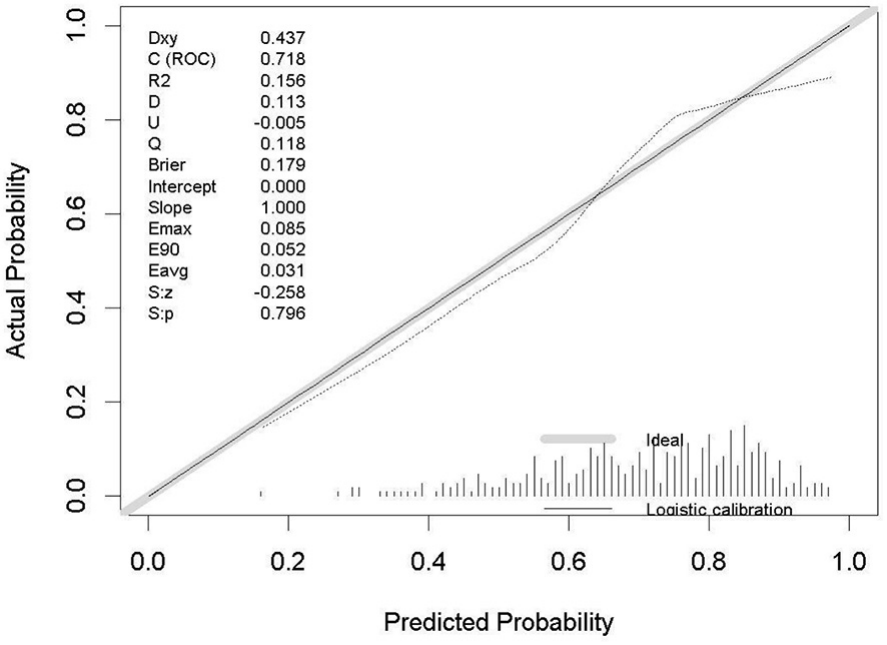
Calibration plot of the multivariable logistic regression model for poor sleep quality.

The multivariable logistic regression model performance was assessed to determine its adequacy, discrimination, calibration, and multicollinearity in predicting poor sleep quality. The model demonstrated a significant improvement over the null model- reduction in deviance from the null deviance (463.84) to the residual deviance (419.12). The model had an Akaike Information Criterion (AIC) of 435.12, suggesting an acceptable balance between model fit and complexity. The Hosmer–Lemeshow goodness-of-fit test showed adequate model calibration (χ^2^ = 8.21, df = 8, *p* = 0.413), indicating good agreement between observed and predicted probabilities of poor sleep quality. Additionally, the calibration assessment using showed excellent calibration performance, with an intercept close to zero (−3.01 × 10^−1^) and a calibration slope of 1.00, indicating minimal evidence of overfitting or underfitting. The Brier score was 0.179, suggesting acceptable overall prediction accuracy. Furthermore, the discriminatory ability of the model was acceptable, with an area under the receiver operating characteristic curve (AUC) of 0.719. Pseudo-R^2^ measures indicated modest explanatory power, with a McFadden's R^2^ of 0.096, Cox and Snell R^2^ of 0.109, and Nagelkerke R^2^ of 0.156, suggesting that the predictors explained approximately 10–16% of the variability in sleep quality status. Finally, Multicollinearity was assessed using variance inflation factors (VIFs). All VIF values ranged from 1.01 to 2.00, indicating no evidence of problematic multicollinearity among the independent variables (Figure 7).

A sensitivity analysis was performed to evaluate the potential influence of overlap between PUMP and SAS-SV scores. In a model including PUMP but excluding SAS-SV, PUMP was not significantly associated with poor sleep quality (OR = 1.01, 95% CI 0.997–1.03, *p* = 0.121). However, SAS-SV remained significantly associated with poor sleep quality when entered without PUMP (OR = 1.03, 95% CI 1.01–1.06, *p* = 0.008). In the full model containing both variables, SAS-SV remained significant (OR = 1.04, 95% CI 1.00–1.07, *p* = 0.027), whereas PUMP remained non-significant (OR = 0.99, 95% CI 0.97–1.02, *p* = 0.640).

## 4 Discussion

### 4.1 Key findings

This cross-sectional study investigated the relationship between problematic smartphone use and sleep quality in 387 Sudanese undergraduate medical students during an ongoing armed conflict. Importantly, higher scores on smartphone addiction (SAS-SV) and problematic use (PUMP) scales were significantly correlated with poorer sleep quality. Chronotype was also associated with sleep outcomes, as a morning preference was protective against poor sleep, while evening tendencies were linked to lower life satisfaction, higher addiction rates and poor sleep quality. Additionally, employment status was significantly associated with poor sleep, with employed students having twice the odds of poor sleep compared to their unemployed peers. Correlation analyses further indicated moderate associations between smartphone addiction, sleep quality, and life satisfaction, suggesting interconnected behavioral and wellbeing patterns among medical students.

### 4.2 Interpretation in the context of existing literature

The study explored the associations between several factors and mobile use, and the extent of smartphone addiction on sleep pattern and quality. Key factors affecting mobile use include ongoing war conflict, academic demands, sleep chronotype, employment status and socio-environmental factors. About half (49%) of participants reported increased smartphone use during the conflict, while 30% reported no increase and 21.9% reported a decrease. The observed smartphone use among nearly half of participants likely reflects increased reliance on mobile devices for social communication, information access, and academic continuity during the conflict. However, this heightened dependence may also extend phone use into nighttime hours and increase exposure to distressing content, thereby contributing to sleep disruption. For example, university students in war-torn Yemen exhibited high rates of smartphone addiction, which researchers attributed in part to limited alternative social resources and the need for connection during ongoing civil conflict (18). Similarly, mobile device adoption in high-risk contexts, such as Afghanistan, remained high despite security concerns, as users prioritized access to information and connectivity (19). Analyses of mobile data after extreme events further demonstrate that phone behavior changes significantly around crises, with both increases and decreases depending on user strategy (19).

Academic responsibilities appear to be a key factor influencing smartphone use among medical students. Smartphones serve essential educational functions, including accessing lecture materials, reviewing notes and checking grades, especially in students with heavy course loads (20). Concurrently, academic stress may be associated with increased non-academic phone use, such as social media, as a coping or avoidance behavior, which can contribute to problematic use and sleep disruption (21). Thus, the dual role of smartphones, as both academic and recreational, helps explain the observed association between higher study demands and increased or more problematic smartphone engagement.

Chronotype emerged as an important factor in the relationship between smartphone use and sleep. Evening-type students may be more likely to engage in late-night phone use, increasing exposure to blue light, cognitive arousal, and time displacement, which together disrupt circadian rhythms and reduce sleep quality (22). This pattern is consistent with research showing that problematic smartphone use occurs more frequently in evening type individuals and is associated with both greater screen time and poorer sleep outcomes across student populations. (23). Conversely, morning-type students were protected from these effects, as reflected in lower odds of poor sleep in our multivariable model (23). These findings align with existing literature demonstrating higher rates of problematic smartphone use and sleep disturbances among evening-type individuals.

Medical students may be especially vulnerable to high rates of problematic smartphone use because these devices serve dual roles as educational tools and constant sources of social and psychological engagement, extending use into periods normally reserved for rest and recovery (24). Excessive nightly smartphone use has been associated with poor sleep quality, including longer sleep latency, difficulty maintaining sleep, and reduced sleep duration, especially when phones are used at bedtime for social media or entertainment (25). This link between higher smartphone addiction scores and compromised sleep is consistently observed in medical student populations globally, with strong positive correlations reported between addiction severity and poor sleep quality indices (26). Poor sleep quality and disrupted sleep patterns have further been associated with lower psychological wellbeing, suggesting that the repercussions of problematic smartphone use extend beyond sleep to affect daytime functioning, mood, and overall quality of life (27).

Due to academic demands and conflict related pressures, psychological and behavioral factors not directly measured in this study, such as stress and anxiety, may further influence smartphone use and sleep. Additional unmeasured factors, including depression, caffeine intake, screen exposure duration, and pre-existing sleep disorders, may also have influenced the observed associations and represent potential sources of residual confounding. Prior research suggests that psychological distress is associated with increased problematic smartphone use and poorer sleep through mechanisms including rumination, hyperarousal, and bedtime procrastination (28, 29).

Two interrelated pathways may explain these findings. First, circadian disruption from prolonged nighttime smartphone exposure may delay melatonin secretion, increase cognitive arousal, and shift sleep timing toward eveningness patterns (9, 10). Second, behavioral addiction mechanisms suggest that compulsive smartphone engagement promotes repetitive nighttime checking, emotional dependence, and difficulty disengaging before sleep, thereby reducing sleep opportunity and impairing sleep quality (30).

A notable finding was the association between employment status and sleep, with employed students showing higher odds of poor sleep; this aligns with prior research indicating that individuals balancing work with academic or daily responsibilities rely more heavily on mobile devices, especially at night, potentially increasing exposure to sleep-disrupting stimuli (31). Finally, socio environmental influences, including limited offline social activities, living situation constraints, and the stressors inherent in conflict-affected environments, have been associated with increased nighttime smartphone engagement, which can further compromise sleep (31, 32).

Although not measured, burnout may serve as a plausible explanatory mechanism linking our variables. Prior evidence suggests that burned-out medical students are more vulnerable to problematic smartphone use and sleep disturbances, with a reported global burnout prevalence of 37.23% (33). Burnout has been associated with academic pressure, workload, financial stress, and psychosocial strain, (34–36). and is consistently associated with poor academic performance, sleep deprivation, psychological distress, and increased risks of depression (34). Burnout-related isolation and stress may be associated with maladaptive coping behaviors, including excessive smartphone use, particularly at night (37). This further contribute to sleep and circadian disruption, potentially contributing to a cyclical association between poor sleep, reduced life satisfaction, and fatigue (38). In conflict-affected and high-demand academic settings, such as the context of this study, burnout may act as an underlying factor that amplifies the observed relationships between excessive smartphone use, evening chronotype tendencies, poor sleep quality, and diminished wellbeing.

### 4.3 Strength and limitations

Our study has several strengths and limitations. First, it is the first study of its kind to examine problematic smartphone use, sleep quality, chronotype, and life satisfaction among Sudanese medical students, thereby filling a critical gap in low-income, conflict-affected settings. Moreover, exploring multiple interconnected domains allowed for a more nuanced analysis. In addition, the study included a relatively large sample size of 387 students, which provided sufficient statistical power for analyses. Furthermore, the use of validated standardized scales (PUMP, SAS-SV, SWLS, PSQI, MEQ) ensured reliability and comparability with other studies. Importantly, multivariable logistic regression identified independent predictors of poor sleep while controlling for confounders such as employment and chronotype. Finally, capturing smartphone use changes during the ongoing Sudanese conflict added a contextually unique real-world perspective and grounded data on environmental influences.

However, several limitations must be acknowledged. Firstly, the cross-sectional design does not allow causal or temporal relationships between smartphone use and sleep disturbances to be established. Secondly, the use of non-probability convenience sampling may limit the representativeness and generalizability of the findings, particularly given the reliance on online recruitment during an ongoing armed conflict. Thirdly, the use of self-reported questionnaires may introduce recall, reporting, and social desirability biases.

Importantly, although the study was conducted during an active armed conflict, we did not directly measure conflict-related exposures or psychological consequences such as trauma exposure, displacement status, direct exposure to violence, PTSD symptoms, or other conflict-related stressors. These unmeasured variables may represent substantial sources of residual confounding, as they could independently influence problematic smartphone use, chronotype, psychological wellbeing, and sleep quality. Therefore, the observed associations should be interpreted cautiously, and the findings should not be understood as reflecting direct effects of smartphone use alone independent of the broader conflict environment.

Additionally, other potentially relevant confounders, including depression, anxiety, caffeine intake, environmental noise, pre-existing sleep disorders, and objective screen-time duration, were not assessed. Finally, some scales, such as the PSQI, demonstrated relatively lower internal consistency in this sample, which may have affected the reliability of sleep-related measurements.

### 4.4 Future research directions

Future research should prioritize longitudinal and interventional designs to clarify the temporal and causal relationships between problematic smartphone use, sleep quality, and wellbeing among medical students. Additionally, interventional studies should test behavioral or educational interventions aimed at reducing excessive phone use and improving sleep hygiene in student populations. Moreover, conflict-specific analyses should investigate the impact of war or ongoing conflict on phone use patterns, sleep, and mental health, using both quantitative and qualitative approaches. Importantly, reducing reliance on self-reported data by including digital usage logs or wearable sleep trackers as objective measures may strengthen findings and decrease bias. Finally, expanding this research to other student populations and low- and middle-income, conflict-affected settings would enhance generalizability and inform context-sensitive interventions.

## 5 Conclusion

Poor sleep quality was highly prevalent among Sudanese medical students during the ongoing armed conflict. Smartphone addiction, evening chronotype, and employment status were significantly associated with poorer sleep quality. However, these findings should be interpreted cautiously because important conflict-related variables, including trauma exposure, displacement, and conflict-associated psychological distress, were not directly measured and may have substantially influenced the observed relationships. Nevertheless, the study highlights the complex interaction between digital behavior, circadian preference, and sleep health within a conflict-affected academic environment. Future longitudinal studies incorporating direct measures of conflict exposure and mental health outcomes are needed to better clarify these relationships and guide targeted interventions.

## Data Availability

The raw data supporting the conclusions of this article will be made available by the authors upon reasonable request.
